# A Case of Bladder Paraganglioma With Negative Photodynamic Diagnosis Completely Resected by Staged En Bloc Transurethral Resection

**DOI:** 10.1002/iju5.70220

**Published:** 2026-07-03

**Authors:** Shu Gozu, Yasukazu Nakanishi, Gaku Okumura, Erika Ikezoe, Naoki Imasato, Kohei Hirose, Madoka Kataoka, Shugo Yajima, Hitoshi Masuda

**Affiliations:** ^1^ Department of Urology National Cancer Center Hospital East Kashiwa‐shi, Chiba Japan

**Keywords:** paraganglioma, photodynamic diagnosis, transurethral resection, urinary bladder, urothelial carcinoma

## Abstract

**Introduction:**

Bladder paraganglioma can be challenging to distinguish from nonpapillary bladder cancer. Here, we report a case of nonfunctional bladder paraganglioma treated with photodynamic diagnosis‐assisted en bloc transurethral resection of the bladder tumor.

**Case Presentation:**

A 78‐year‐old man was incidentally diagnosed with a bladder tumor on imaging. Radiological and cystoscopic findings suggested bladder paraganglioma or nonpapillary urothelial carcinoma. The patient underwent en bloc transurethral resection of the bladder tumor with photodynamic diagnosis using 5‐aminolevulinic acid. No fluorescence enhancement was observed during photodynamic diagnosis. Histopathological examination revealed nonfunctional bladder paraganglioma. A second‐look en bloc transurethral resection with photodynamic diagnosis confirmed the absence of a residual tumor.

**Conclusion:**

This case highlights the diagnostic pitfalls of nonfunctional bladder paraganglioma presenting as a photodynamic diagnosis‐negative bladder tumor and suggests that en bloc transurethral resection may be an effective organ‐preserving approach in selected cases.

## Keynote Message

Nonfunctional bladder paraganglioma is a rare tumor that closely mimics urothelial carcinoma on imaging and cystoscopy. Photodynamic diagnosis using 5‐aminolevulinic acid may yield false‐negative results for non‐urothelial tumors. En bloc transurethral resection allows for accurate pathological assessment and may provide adequate local control in selected patients.

## 1 Introduction

Paragangliomas are rare neuroendocrine tumors arising from extra‐adrenal chromaffin cells and account for approximately 10%–15% of pheochromocytomas–paraganglioma syndromes [1]. The urinary bladder is an uncommon site, representing less than 0.05% of all bladder tumors and approximately 6%–10% of extra‐adrenal paragangliomas [2]. Bladder paragangliomas (BPG) are classified as functional or nonfunctional according to catecholamine secretion. Functional tumors typically present with hypertension, palpitations, headache, diaphoresis, and micturition‐induced hypertensive crises. In contrast, nonfunctional tumors lack adrenergic symptoms and are frequently detected incidentally or misdiagnosed as urothelial carcinoma. Moreover, hematuria is the most common presenting symptom but is nonspecific [3]. Cross‐sectional imaging often demonstrates a hypervascular intravesical mass, and magnetic resonance imaging (MRI) findings may resemble urothelial carcinoma [4, 5]. Although the Vesical Imaging‐Reporting and Data System (VI‐RADS) is useful for staging urothelial carcinoma, it does not commonly adopted nonurothelial tumors. Urinary cytology is usually negative because of the nonepithelial origin of the tumor [5]. Photodynamic diagnosis (PDD) using 5‐aminolevulinic acid or hexaminolevulinate improves detection of non‐muscle‐invasive urothelial carcinoma and reduces residual tumor rates [6]. However, its diagnostic value in nonurothelial tumors remains unclear. Few reports have described PDD findings in patients with BPG. Here, we report a case of nonfunctional BPG that was negative for PDD and was managed by staged en bloc transurethral resection of the bladder tumor (ERBT).

## 2 Case Presentation

A 78‐year‐old man was referred to our institution for further evaluation of a bladder mass that had been incidentally detected at another hospital on contrast‐enhanced computed tomography (CT) performed during a workup for low back pain. CT revealed a 14‐mm, well‐demarcated intraluminal mass on the left lateral bladder wall with early arterial enhancement and no evidence of extravesical extension (Figure 1A). No upper urinary tract tumors, lymph node metastases, or distant metastases were identified. His medical history included hypertension without palpitations, and his surgical history included remote duodenal ulcer surgery via midline laparotomy. Pelvic magnetic resonance imaging (MRI) revealed a T2‐hyperintense lesion confined to the bladder wall (Figure 1B). Diffusion‐weighted imaging demonstrated a focal high‐signal‐intensity lesion corresponding to the tumor (Figure 1C). Based on these findings, the lesion was categorized as VI‐RADS category 3.

**FIGURE 1:**
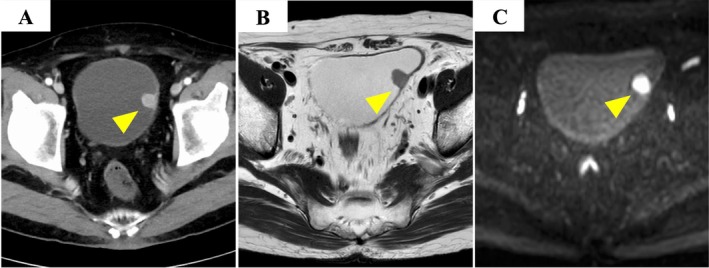
Radiologic findings of the bladder tumor. (A) Contrast‐enhanced computed tomography showing a well‐demarcated hypervascular intraluminal mass on the left lateral bladder wall. (B) Magnetic resonance imaging demonstrating a hyperintense lesion on T2‐weighted imaging. (C) Diffusion‐weighted magnetic resonance imaging showing a focal high‐signal‐intensity lesion corresponding to the tumor.

Cystoscopy revealed a sessile tumor, approximately 20‐mm in diameter, on the left lateral wall of the bladder (Figure 2A). Urinary cytology was classified as class II. Although the imaging findings were not entirely characteristic of bladder paraganglioma, the lesion demonstrated early arterial enhancement on contrast‐enhanced CT and a hypervascular sessile appearance on cystoscopy. Therefore, bladder paraganglioma was included in the differential diagnosis together with nonpapillary urothelial carcinoma. Plasma catecholamines and urinary vanillylmandelic acid (VMA) and homovanillic acid (HVA) levels were measured preoperatively. Plasma catecholamine levels were within institutional reference ranges (adrenaline, 91 pg/mL; noradrenaline, 613 pg/mL; dopamine, 11 pg/mL). Urinary VMA and HVA levels were within normal limits, suggesting a nonfunctional tumor. Under spinal anesthesia, ERBT was performed using oral 5‐aminolevulinic acid–assisted PDD. Although PDD was used for tumor visualization and margin delineation, no fluorescence enhancement was observed under blue light (Figure 2B,C). Standard intraoperative monitoring (noninvasive blood pressure cuff, electrocardiography, and pulse oximetry) showed no hypertensive episodes, tachycardia, or hemodynamic instability, and the procedure was uneventfully completed. Histopathological examination demonstrated a paraganglioma characterized by a nested “Zellballen” architecture (Figure 3A). Immunohistochemical staining was positive for chromogranin A (Figure 3B), synaptophysin (Figure 3C), and CD56, with sustentacular cells highlighted by S‐100 staining (Figure 3D). The patient tested negative for cytokeratin. The Ki‐67 labeling index was < 1%. The adrenal pheochromocytoma and paraganglioma (GAPP) score was 0, which was consistent with a well‐differentiated type. No invasion of the muscularis propria was identified, and the horizontal margin status was indeterminate. A second ERBT with PDD was performed. No residual tumors were histologically identified. The postoperative course was uneventful. Three months postoperatively, no recurrence was observed.

**FIGURE 2:**
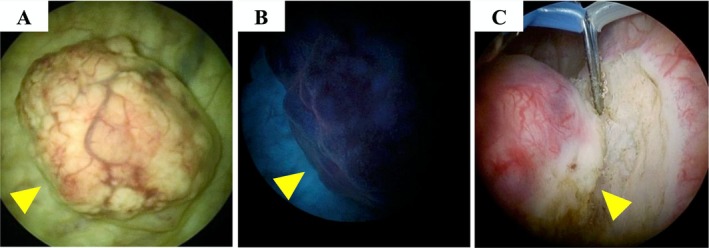
Endoscopic findings with photodynamic diagnosis. (A) White‐light cystoscopy revealing a sessile tumor. (B) Blue‐light cystoscopy following oral 5‐aminolevulinic acid administration showing no fluorescence enhancement. (C) En bloc transurethral resection of the bladder tumor (ERBT).

**FIGURE 3:**
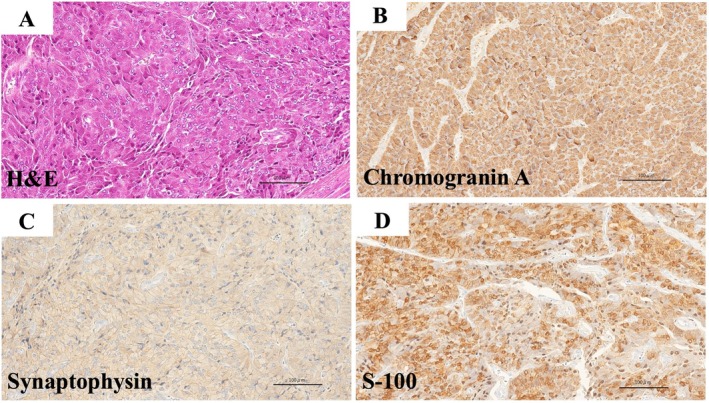
Histopathological findings of the bladder tumor (scale bar = 100 μm). (A) Hematoxylin and eosin staining showing a characteristic nested “Zellballen” pattern. (B) Tumor cells positive for chromogranin A. (C) Tumor cells positive for synaptophysin. (D) S‐100 staining highlighting sustentacular cells.

## 3 Discussion

BPG is often difficult to diagnose preoperatively because of its rarity and variable clinical presentation, particularly in nonfunctional tumors lacking catecholamine‐related symptoms [3]. Measurement of plasma or urinary free normetanephrine and metanephrine levels is recommended as the first‐line screening test; however, these assays were not performed because they are not available at our institution. In the present case, biochemical evaluation confirmed a nonfunctional tumor without catecholamine excess. Although bladder paraganglioma was included in the differential diagnosis because of the hypervascular appearance on imaging and cystoscopy, additional functional imaging studies such as MIBG scintigraphy were not performed. Given the absence of catecholamine‐related symptoms and the small localized nature of the lesion, diagnostic and therapeutic ERBT was prioritized. The hypervascularity and atypical endoscopic appearance nevertheless raised suspicion of a nonurothelial tumor.

Radiologically, BPG typically appears as a hypervascular intravesical mass on contrast‐enhanced CT and demonstrates T2 hyperintensity with early enhancement on MRI [4, 5]. These imaging features overlap with those of urothelial carcinomas. A distinctive aspect of the present case was the repeated absence of fluorescence in 5‐aminolevulinic acid‐assisted PDD. Although PDD improves tumor detection and reduces residual tumor rates in non‐muscle‐invasive bladder cancer [6], its diagnostic value for nonurothelial tumors remains undefined. Consistent PDD‐negative findings may reflect limited protoporphyrin IX accumulation in paraganglioma cells; however, their diagnostic implications remain uncertain.

In a 25‐year systematic review including 199 patients, Liu et al. reported that transurethral resection (TUR) was performed in 53.3% of cases, partial cystectomy in 39.7%, and radical cystectomy in 2.5%. Regarding surgical options, partial cystectomy has been mainly performed for preoperatively diagnosed BPG. Surgical approach differed according to preoperative diagnostic accuracy [7]. However, accumulating evidence indicates that transurethral approaches may be adequate for small, localized, and nonfunctional tumors. Xu et al. reported successful management of a nonfunctional BPG via TUR without recurrence [8]. Nationwide data also demonstrated that TUR was safe and effective for tumors ≤ 3 cm when complete excision was achieved [9]. Matsumoto et al. described successful complete resection using en‐bloc resection of bladder tumor (ERBT) [10]. Similarly, Zhu et al. reported successful management using transurethral holmium laser resection [11]. ERBT avoids tumor fragmentation and enables precise pathological evaluation. In the present case, although the initial specimen demonstrated an indeterminate horizontal margin, a planned second ERBT confirmed complete tumor removal. More than half of BPGs are misdiagnosed preoperatively, and such misdiagnoses are associated with increased intraoperative complications [7]. These findings emphasize the importance of biochemical screening and perioperative planning.

Metastatic disease from BPG has been reported in 9% to 48% of cases [2]. Therefore, long‐term surveillance is required. In our patient, the tumor was small, nonfunctional, completely resected after staged ERBT, and demonstrated low proliferative activity (Ki‐67 < 1%). The patient remained recurrence‐free. In conclusion, complete transurethral en bloc resection can be curative in selected small, localized, nonfunctional tumors.

## Ethics Statement

The protocol for this study was approved by the Ethics Committee of National Cancer Center Hospital East (Approval No. 2018‐159).

## Consent

Written informed consent was obtained from the patient for publication of this case report and accompanying images.

## Conflicts of Interest

The authors declare no conflicts of interest.

## Data Availability

The data that support the findings of this study are available on request from the corresponding author. The data are not publicly available due to privacy or ethical restrictions.
